# SARS-CoV-2 versus Influenza A Virus: Characteristics and Co-Treatments

**DOI:** 10.3390/microorganisms11030580

**Published:** 2023-02-24

**Authors:** Xinyi Wang, Jingwen Li, Hanshu Liu, Xinyu Hu, Zhicheng Lin, Nian Xiong

**Affiliations:** 1 Department of Neurology, Union Hospital, Tongji Medical College, Huazhong University of Science and Technology, Wuhan 430000, China; 2 Mclean Hospital, Harvard Medical School, Belmont, MA 02478, USA

**Keywords:** SARS-CoV-2, influenza A, co-treatments, variants, smoking and alcohol use

## Abstract

For three years, the novel coronavirus disease 2019 (COVID-19) pandemic, caused by infection of the SARS-CoV-2 virus, has completely changed our lifestyles and prepared us to live with this novel pneumonia for years to come. Given that pre-existing flu is caused by the influenza A virus, we have begun unprecedently co-coping with two different respiratory diseases at the same time. Hence, we draw a comparison between SARS-CoV-2 and influenza A virus based on the general characteristics, especially the main variants’ history and the distribution of the two viruses. SARS-CoV-2 appeared to mutate more frequently and independently of locations than the influenza A virus. Furthermore, we reviewed present clinical trials on combined management against COVID-19 and influenza in order to explore better solutions against both at the same time.

## 1. Introduction

In late January 2020, news flooded the world about the outbreak of novel coronavirus disease 2019 (COVID-19). Swift spread of this new infectious disease disturbed people all over the world. On 11 March 2020, the World Health Organization (WHO) declared the pandemic caused by a novel coronavirus, renamed severe acute respiratory syndrome coronavirus 2 (SARS-CoV-2), as a public health emergency of international concern. Afterwards, this outbreak quickly caused enormous human casualties and global economic losses, which then aroused global panic [1]. Currently, with the development of various COVID-19 vaccines, achieving global vaccine coverage has been considered as the major measure to control the COVID-19 pandemic, providing protection against severe disease and hospitalization from COVID-19. However, a new heavily mutated SARS-CoV-2 variant known as Omicron has aroused widespread concern on the new waves of infection, with increased transmissibility and a higher risk of reinfection than before [2]. The substantially increasing numbers of infections with the Omicron variant around the world have suggested that the COVID-19 pandemic is far from over [3,4].

Previously, the world has been challenged with four pandemics caused by various strains of another RNA virus, the influenza virus [5]. Over the past hundred years, influenza A viruses have been the major pathogen causing multiple global outbreaks of acute respiratory infections, resulting in approximately 50 to 100 million of the world’s population dying [6]. The most recent influenza pandemic was caused by the 2009 H1N1 influenza A virus, which has remained with us and is known as annual seasonal influenza. Taking the continuous antigenic mutations of the influenza virus into consideration, we presume that the appearance of a new influenza pandemic is highly inevitable [7].

To better understand the characteristics of and simultaneously cope with the two pandemics, we have performed a comparative study on SARS-CoV-2 vs. influenza A virus, regarding the virology, epidemiology, immune response mechanism and the main variants of the two viruses (Table 1). Environmental risk, such as tobacco and alcohol use, is considered too. Furthermore, clinical trials on co-intervention against seasonal flu and COVID-19 are reviewed to provide ongoing efforts for co-intervention.

## 2. Virology of Influenza A Virus and SARS-CoV-2

Both influenza A virus and SARS-CoV-2 caused the global pandemics, leading to significant health and economic threats. Here, we compare the virology of the two viruses, including the structure, genome, origin, reservoir, virus entry and genetic mutation.

### 2.1. Influenza A Virus

Influenza A virus, an enveloped, negative-sense and single-stranded RNA virus, belongs to the family Orthomyxoviridae [5]. Influenza A virus has a genome of eight different segments encoding eighteen different proteins with specific functions, including the envelope proteins hemagglutinin (HA) and neuraminidase (NA), acidic polymerase proteins (PA) and basic polymerase proteins (PB), nucleoprotein (NP), matrix proteins (M) and non-structural proteins (NS) [7]. Among them, HA and NA play a key role in the viral attachment to the cellular receptor and release from host cells, respectively, which are also the primary targets of the neutralizing antibodies [23]. Moreover, influenza A virus is classified by surface HA and NA glycoproteins, which contain 16 different HA and 9 different NA sub-types [24]. According to the observations of influenza pandemics in the 20th century, only the H1, H2 and H3 sub-types of influenza A viruses have spread among human as huge threats to global health (human infections from poultry-adapted influenza A viruses such as H5N1 and H7N9 excepted) [23]. Wild waterfowl has been known for some time to be the natural reservoir of all influenza A viruses [25]. Human infections with influenza A viruses are associated with host-switching events occurring in wild waterfowl virus. Specifically, a waterfowl virus can adapt to new mammals or the human hosts via accumulating point mutations or reassortment with gene segments from a different influenza virus, capable of infecting human respiratory epithelial cells and spreading within the population [26,27].

The first step for influenza A virus entry into the body is the binding of HA to the terminal glycosides of sialic acid on the surface of respiratory epithelial cells, which initiates the viral endocytosis, followed by the ribonucleic acid (RNA) replication and release from host cells [17]. Influenza A virus displays a high capacity for antigenic variation, allowing for the accumulation of single nucleotide mutations in the viral genes because the reverse transcription polymerase lacks effective function of proofreading the bases [5]. Small changes in envelope glycoproteins due to point mutations in the HA and NA genes over time are defined as “antigenic drift”, while “antigenic shift” is due to gene reassortment from different influenza virus strains, which may cause a novel global pandemic due to a lack of population immunity to the new strain [28].

### 2.2. SARS-CoV-2

SARS-CoV-2 was first documented at the end of 2019, and its origin remains debatable [29,30]. Previous sequence analysis showed that SARS-CoV-2 belongs to the B lineage of the genus β-CoVs in the family of Coronaviridae, which is an enveloped, single-stranded, positive-sense RNA virus with a genome size of 29.9 kb, the largest genome among RNA viruses [31,32]. Previous studies reported that the SARS-CoV-2 genome contains 14 open reading frames (ORFs) that encode about 27 proteins, including spike (S), envelope (E), membrane (M) and nucleocapsid (N) proteins [7,33]. In addition, a metagenomics analysis indicated that SARS-CoV-2 shared 79.5% sequence identity with SARS-CoV, which caused the outbreak of SARS 20 years ago. In contrast, Shi et al. reported that there was a 96% similarity of the whole genome shown between SARS-CoV-2 and a bat CoV [34], hence the bats were considered as the probable nature reservoir of SARS-CoV-2 [13]. Moreover, pangolins were reported as the possible intermediate hosts which played a key zoonotic role in the animal-to-human transmission [35]. When SARS-CoV-2 invades host cells, the S proteins bind to angiotensin converting enzyme 2 (ACE2) receptors which are highly expressed on alveolar type Ⅱ epithelial cells, with the cleavage of S protein mediated by transmembrane serine proteases 2 and 11D (TMPRSS2, TMPRSS11D), allowing for fusion between virus and cell membranes, and then virus entry [7,36]. Accumulating evidence suggested that missense and synonymous mutations were considered as the most common mutations for the SARS-CoV-2 genome [33]. Furthermore, particular attention was directed to the mutations of the S protein which was the main antigen inducing the neutralization antibody after SARS-CoV-2 infection [20,37].

## 3. Epidemiology of Influenza A Virus and SARS-CoV-2

According to the published literature, SARS-CoV-2 is similar to influenza A virus in its route of transmission. Current evidence has shown that possible human-to-human transmission routes of COVID-19 and influenza A include respiratory droplet, aerosol transmission and direct contact with contaminated surfaces [38,39]. Thus, people around a patient coughing, sneezing or talking with others were under a high risk of being infected. Additionally, the positive specimens could be detected in the feces of people infected with COVID-19 or influenza A, suggesting its possibility to spread via the fecal-oral route too [24,40]. In general, all body fluids and secretions from confirmed cases are considered infectious [24,41]. Influenza A affects primarily young healthy adults, with a median age of 12 to 17 years [28]. In contrast, the vulnerable populations of COVID-19 infection mainly included older adults and those in comorbid conditions, with the increased risk of severe cases [42]. As reported, patients infected with influenza A frequently develop symptoms in a period of 1–7 days of exposure and become infectious about 1 day before the onset of symptoms [43,44]. Notably, 80% of patients may still be infectious within 5 days after symptoms resolve [28]. However, the data from previous reports have indicated that the median incubation period of COVID-19 is about 5 days, 97.5% of all infections will develop symptoms within 11.5 days [45]. Notably, a study has reported an unusual COVID-19 case with a 24-day incubation period [46]. It is reported that over one-third of people around the world were infected with the 1918 influenza virus and estimated deaths due to the 1918 influenza epidemic ranged from 50 to 100 million [14,25]. In contrast, as of 24 January 2023, the WHO reported that there have been around 0.6 billion confirmed cases, including 6.7 million deaths from COVID-19. Currently, vaccination campaigns throughout the world are considered to be the most efficient way to fight the COVID-19 pandemic through developing a herd immunity [47].

## 4. Host Immune Response of Influenza A Virus and SARS-CoV-2

### 4.1. Innate Immune Response

As an RNA virus, the SARS-CoV-2 innate immune response is similar to that of influenza A virus [48,49]. The innate immune system, mainly mediated by interferon and as the first line of defense against virus infection, is characterized by quick reaction but the lack of specific effects and memory [48,49,50]. When virus infection initially occurs in the upper respiratory tract, mucus barriers serve as the first protective layer, consisting of various defensive compounds, such as mucins and lysozyme secreted by mucosal epithelial cells [51]. After the virus crosses the mucus barriers, pathogen-associated molecular patterns (PAMPs), containing molecular structures that are found in microbes but not in hosts, are identified by the host’s pattern recognition receptors (PRRs) including toll-like receptor (TLR), retinoic acid-inducible gene I (RIG-I) and melanoma differentiation-associated gene 5 (MDA5) [52]. Subsequently, the high expression of various transcription factors including interferon regulatory factor (IRF) 3 and IRF7 and nuclear factor kappa-light-chain-enhancer of activated B cells (NF-κB) could be observed in the host cells, with abundant expression of interferons and inflammatory cytokines, such as type Ⅰ/Ⅲ interferon, tumor necrosis factor alpha (TNF-α), interleukin (IL)-1 and IL-6 [53,54]. Among them, type I interferon plays an important role in enhancing the antiviral activity of innate immune response [55]. The release of various cytokines accompanied by rapid recruitment of innate effector cells in the infection site, such as monocytes, macrophages, neutrophils and natural killer (NK) cells, limits early viral spread and subsequently facilitates the development of adaptive immune response [49,56].

### 4.2. Adaptive Immune Response

The adaptive immune system develops a highly specific response in the host body, responsible to viral clearance and establishment of immune memory [50]. Previous studies have indicated that CD4^+^ T and CD8^+^ T cells and B cells play a key role in adaptive immunity against virus infection [48,49]. In the influenza A infection, the secretion of interferons in the innate immune response induces the maturation of dendritic cells (DCs) which serve as the antigen-presenting cells (APCs) [48]. Subsequently, viral antigenic peptides are detected by major histocompatibility complex (MHC) class Ⅰ/Ⅱ molecules on the activated APCs and presented to adaptive T lymphocytes via the binding of (MHC) class Ⅰ/Ⅱ molecules with T-cell receptor (TCR) [48,50], facilitating the activation of naïve cluster of differentiation (CD) 4^+^ and CD8^+^ T cells [49]. Specifically, through MHC class Ⅰ molecule binding, CD8^+^ T cells differentiate into cytotoxic T lymphocytes (CTLs) which produce cytotoxic granules (including perforin and granzymes) and cytokines (e.g., interferon-γ and TNF-α) to induce apoptosis of the virus-infected cells [48]. CD4^+^ T cells primarily differentiate into T help 1 (Th1) cells which promote the secretion of antiviral cytokines, such as interferon-γ and IL-2, capable of inhibiting the virus replication [50]. In addition, CD4^+^ T cells can differentiate into Th2, Th17, regulatory T (Treg) cells and follicular helper T (Tfh) cells [57]. Among them, Tfh cells play an important role in promoting B cells to produce antibodies by directing the formation of germinal centers and providing multiple co-stimulatory signals [58]. CD4^+^ T cells also assist the generation of effector CD8^+^ T cells as a result of licensing APCs via the CD40L-CD40 interactions [59,60]. Moreover, non-neutralizing antibodies generated by B cells are indispensable for viral elimination, and influenza A virus-specific antibodies directed to the HA and NA are associated with protective immunity, with a neutralizing ability [61].

In contrast, the adaptive immune response to SARS-CoV-2 infection is not fully understood. The present data indicated that SARS-CoV-2 infection is characterized by increased neutrophil counts and significant lymphopenia, in particular severe COVID-19 pneumonia [62]. Lymphopenia implies that the depletion of both CD4^+^ and CD8^+^ T cells may be associated with virus-induced inhibition of the type I interferon, leading to poor effector T cells response, especially CD8^+^ T cells’ [49,54,63]. In addition, the humoral immune response to SARS-CoV-2 infection is mediated by antibodies against viral surface glycoproteins—the S proteins and N proteins [64,65]. Previous studies have shown that these antibodies are detectable about 14 days after SARS-CoV-2 infection [66]. The neutralizing antibodies directed to the receptor-binding domain (RBD) of the S protein are capable of viral clearance and preventing re-infection [67].

## 5. Evolution of Influenza A Virus and SARS-CoV-2

### 5.1. Variants and Sub-Types of Influenza A Virus

**Figure 1:**
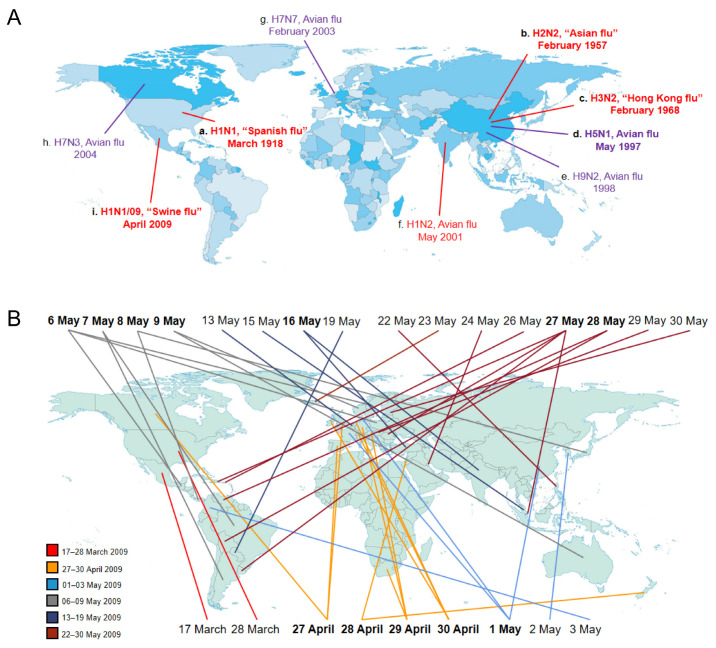
Chronological distribution of influenza A variants and sub-types. (**A**) Human flu viruses (in red) and five avian flu viruses (in purple) capable of infecting humans. The different colors on the world map (from light to deep blue) are here to show the different geographical areas or countries. The time when the virus first appeared in this world is mapped out. Lowercase letters (a–i) display the order in which the influenza A viruses appeared. The four human flu viruses (H1N1, H2N2, H3N2, 2009 H1N1) in bold indicate that they have caused global pandemics, posing a serious threat to human health. H5N1 avian flu virus in bold suggests that it arouses global concern as a potential pandemic threat. (**B**) The first reporting of 2009 H1N1 virus. The first confirmed cases were documented in Mexico on 17 March 2009. Then, the human outbreak of 2009 H1N1 virus rapidly spread to other countries within 2–3 months (from 17 March to 30 May 2009). The six colors of lines stand for different time periods individually. The twelve dates in bold represent that the virus was simultaneously showing up in two or more places in the world.

Influenza A viruses are categorized into sub-types based on the type of two proteins on the surface of the viral envelope—HA and NA glycoproteins. Different influenza viruses encode for different HA and NA proteins. There are 16 known types of HA and 9 known types of NA. Variants are sometimes named according to the host, which mainly include human flu virus and avian flu virus. As reported, only the H1, H2 and H3 sub-types of influenza A viruses have been confirmed to spread in humans, which are called “human flu virus”, such as H1N1, H1N2, H2N2 and H3N2 strains. In addition, some isolates of influenza A virus cause severe disease in domestic poultry, rarely in humans, which are called “avian flu virus”, such as H5N1, H7N3, H7N7, H7N9 and H9N2 strains (Figure 1A). China saw three flu pandemics within 40 years, and North America had two in 90 years. In this review, we are concerned primarily with human flu virus, especially the influenza A viruses causing human influenza pandemics.

Since 1918, influenza A viruses have caused four human pandemics, suggesting that influenza viruses pose a continual threat to global health. The 1918 influenza A pandemic, known as “Spanish influenza”, was caused by an H1N1 influenza A virus strain. The earliest documented case was March 1918 in Kansas, United States (USA). Until now, convincing evidence supporting particular locations of pandemic origin are lacking [25]. As reported, this pandemic infected approximately 500 million people and led to over 50 million deaths, with high pathogenicity and youth-specific mortality [5,27]. The mortality rate of the European population is about 1.1%, considerably higher than the mortality rate in the USA. Previous data in regard to the 1918 pandemic indicated that the majority of symptomatic patients presented typical influenza and recovered after about 7 days, while a small group still died from severe pneumonia as a result of the lower respiratory involvement [25]. The origin of the 1918 pandemic virus remains unknown. The analyses of complete 1918 influenza virus coding sequence have indicated that it was likely derived from a wild waterfowl influenza A virus which became a new virus strain adapting to spread in humans through accumulating point mutations [25,68,69]. Since that time, the 1918 H1N1 influenza A virus as a “founder virus” has initiated a new era of influenza A virus pandemic. The subsequent influenza A pandemics and seasonal epidemics have been caused by descendants of the 1918 pandemic virus, including the 1957 “Asian influenza” by H2N2, the 1968 “Hong Kong flu” by H3N2 and the 2009 “swine flu” by a novel H1N1 virus [27]. The descendants of the 1918 H1N1 virus all showed an obviously decreased virulence, with relatively lower mortality [26,27].

In 1957, “Asian influenza” caused by an H2N2 influenza A virus strain led to almost 1–4 million deaths worldwide, which displaced the 1918 H1N1 virus and spread among human from 1957 to 1968. In 2013, the WHO estimated the case fatality rate (CFR) of Asian flu to be lower than 0.2%. As reported, with the exception of HA, NA and PB1 segments, the remaining segments in the H2N2 genome were preserved from the 1918 H1N1 strains [23]. Moreover, the sequence analysis indicated that both H2 and N2 segments were very similar to avian-derived sequences, with only slight differences in several amino acids, suggesting that the H2N2 strain was likely generated by reassortment between the 1918 H1N1 virus and circulating avian influenza virus strains [23,26,70]. In 1968, the H3N2 strain first emerged in Hong Kong, China and completely replaced the H2N2 strain, leading to about 1 million deaths worldwide [5]. As reported, the death rate from the Hong Kong flu was lower than most other 20th century pandemics. Additionally, the phylogenetic analyses indicated that the H3 segment most likely derived from an avian source [23]. The most recent influenza A pandemic was caused by a novel H1N1 influenza A virus in 2009, causing about 0.28 million deaths. The first human outbreak of 2009 H1N1 occurred in Mexico in March 2009, quickly spreading to 62 counties within 2–3 months (from 17 March 2009 to 30 May 2009) [71]. The times that the first announcements of confirmed cases were reported by the countries are marked on the map, suggesting that this virus might mutate independently of locations per the 27 May reports (Figure 1B). The 2009 H1N1 influenza A virus, a reassortment virus, comprises four different genetic elements from classical swine, Eurasian swine, human and avian strains of influenza A [72]. In addition, the pathogenicity of the 2009 H1N1 virus was remarkably weaker than that of the 1918 pandemic virus, which has been spreading as a seasonal flu virus since that time. Notably, the 2009 H1N1 influenza virus did not replace the circulating H3N2 strain that has been spreading in humans [70]. Thereby, both the 2009 H1N1 influenza virus and the H3N2 virus strain have been co-circulating in humans since 2009.

### 5.2. The Variants of SARS-CoV-2

**Figure 2:**
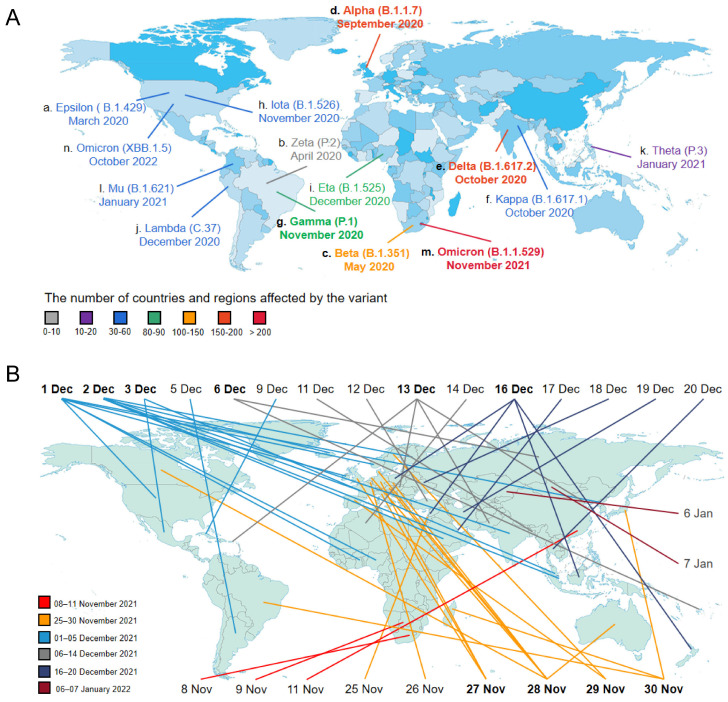
Chronological distribution of SARS-CoV-2 variants. (**A**) SARS-CoV-2 evolution. Different countries are presented via the colors on the world map (from light to deep blue). We delineate the time when different SARS-CoV-2 variants first appeared in this world on this map, displaying months using 3-letter acronyms. The figure uses letters of the Greek alphabet for naming variants of interest (VOIs) and variants of concern (VOCs), with Pango lineage for naming SARS-CoV-2 genetic lineages. Lowercase letters (a–n) display the order in which the SARS-CoV-2 variants appeared. The seven colors of line and font each represent the number of countries affected by the variant. The five variants in bold are currently circulating VOC (Omicron) and previously circulating VOCs (Alpha, Beta, Gamma and Delta). (**B**) SARS-CoV-2 Omicron variant’s first reporting. According to the WHO, Omicron variant, as the only currently circulating VOC, was first documented in South Africa on 8 November 2021, which continued to sweep the world within 2–3 months (November 2021–January 2022). The six colors of line stand for different time periods individually. The ten dates in bold represent that Omicron variant was simultaneously showing up in two or more places in the world.

Accumulating evidence suggested that the more frequently SARS-CoV-2 virus spreads, the higher the viral mutation rates that can occur. In the start of the COVID-19 pandemic, the possibility of mutations in the SARS-CoV-2 genome was relatively low, with limited numbers of people infected. However, due to huge numbers of infections and long-term infections in immunocompromised patients, the variations of SARS-CoV-2 have been continuously appearing [73]. During late 2020, the WHO recommended the classification of novel SARS-CoV-2 strains as variants of interest (VOIs) and variants of concern (VOCs) [74]. VOIs contain genetic changes that are predicted to alter virus characteristics such as transmissibility, disease severity and immune escape, with significant community transmission or international spread. A VOC is defined as a virus that carries multiple mutations compared with the reference genome, with powerful evidence to support the increased transmissibility, virulence or a decrease in the response to vaccines and therapies [75,76]. The first report of each new variant is now indicated in the world map (Figure 2A). According to this map, a dozen different variants of SARS-CoV-2 appeared in the world within one year, indicating that the viral genome mutated rapidly. We also found that more than one variant occurred in the same country, such as three different variants in the USA, and two different variants in Brazil, India and South Africa. We postulated that this might be related to the wide spread of COVID-19 infection, resulting from a poor COVID-19 vaccination rates and inadequate preventive measures in these countries. Therefore, reasonable precautions and expanding vaccine coverage are extremely important to prevent the mutations of the virus and to effectively control the COVID-19 pandemic. In addition, at the time of this writing, Epsilon (B.1.427 and B.1.429), Zeta (P.2), Kappa (B.1.617.1), Iota (B.1.526), Mu (B.1.621), Eta (B.1.525), Lambda (C.37) and Theta (P.3) are considered as previously circulating VOIs, due to the steep decline in circulation of the variants. Likewise, Alpha (B.1.1.7), Beta (B.1.351.1), Delta (B.1.617.2) and Gamma (P.1) are designated as previously circulating VOCs, which have been demonstrated to no longer pose a major added risk to global public health compared to other circulating SARS-CoV-2 variants such as Omicron (B.1.1.529), a variant first detected in South Africa.

The Alpha variant, also known as B.1.1.7, was initially identified in the United Kingdom (UK) in September 2020, which might be associated with the prolonged infection of an immunocompromised host [77]. Compared to the reference strain, it has eight key S protein mutations, three of which play a key role in increasing the infectivity of B.1.1.7, including N501Y, H69/V70 deletion and P681H/R. The N501Y mutation occurs in the RBD of the S protein, responsible for promoting the virus binding to the host ACE2 receptors [78]. The Beta variant, also known as B.1.351, was first detected in South Africa in May 2020 and contains nine mutations in the S protein. Among the nine spike mutations, the N501Y (shared with Alpha variant), E484K and K417N mutations are in the RBD, resulting in the enhanced binding affinity for ACE2 and immune evasion of the virus [79]. In November 2020, the Gamma variant, also known as P.1, is a branch of the B.1.1.28 lineage which was first documented in Brazil. It contains 12 mutations in S gene, including three RBD mutations—N501Y (shared with the Alpha and Beta variants), E484K (shared with the Beta variants), K417T and five amino-terminal domain (NTD) mutations (L18F shared with Beta) [80]. Both RBD and NTD in the S protein are the targets of neutralizing antibodies, implying that a high portion of monoclonal antibodies, including these with the Food and Drug Administration (FDA) Emergency Use Authorizations (EUA), might fail to efficiently neutralize the P.1 variant [78,81]. The Delta variant, also known as B.1.617.2, was first identified in Maharashtra, India on the 5 October 2020. B.1.617.2 has seven mutations in the S protein, including P681R shared with the alpha variant, and two different mutations within the RBD (L452R, T478K) which are associated with increased transmission and reduced antibody neutralization [77]. As reported, Delta is more contagious compared with other previously known variants [82], leading to the second wave of massive infections in India, as well as rapidly replacing the Alpha variant in the UK and USA [80]. As the time of writing this article, the above four SARS-CoV-2 variants have been considered as previously circulating VOCs by the WHO as a result of no longer posing a major added risk to global public health.

On 26 November 2021, a new variant named Omicron (B.1.1.529) has been designated as a VOC by the WHO, raising public concerns on the new wave of infection due to its high infectivity and partial vaccine escape [83]. Omicron initially was identified in South Africa on 21 November 2021, and then rapidly swept to 208 countries, becoming the globally dominant variant as well as the only currently circulating VOC [84]. As shown in Figure 2B, Omicron variant spread rapidly around the world within 2–3 months (November 2021–January 2022), indicating that this virus could mutate simultaneously at various locations, as per the data for 27 November–3 December and 13 and 16 December. The variant was reported to appear in different countries on the same day. For example, on 2 December 2021, Omicron was first identified in seven different countries, which might be related to a recent travel history to the area of Omicron variant (such as South Africa and Nigeria) or indicate independent mutations. According to the African Medical Association, Omicron is seven times more infectious than the Delta variant, but the number of reported deaths in Africa has continued to decline, indicating that the new variant developed a lower virulence [85]. As reported, the number of mutations in the genome of the Omicron variant is significantly larger than that of other VOCs [77]. Notably, 32 mutations have been identified in the S protein which the virus utilizes to enter host cells and is targeted by neutralizing antibodies [86]. Fifteen of these mutations locate at RBD of the S protein, which are associated with the higher affinity to the ACE2, leading to increased infectivity and partial resistance to the neutralizing antibody induced by COVID-19 vaccines [87]. Recent studies have indicated that the Omicron variant has caused an increase in cases of reinfections around the world, in particular in South Africa where only 7.5% of people have been vaccinated [88,89]. Increasing reports of breakthrough infections caused by Omicron variants have aroused public concerns about the efficacy of existing vaccines [86]. The present findings have suggested that two doses of vaccine provide limited protection against symptomatic infection caused by the Omicron variant because protection wanes over time [90,91,92]. Thereby, a third or a booster dose of vaccine have been advised to increase the neutralizing activity of the Omicron variant [93]. Accumulating evidence from case–control studies implicated that three doses of mRNA vaccine significantly increased protection against the Omicron variant [91,94]. In addition, effective containment of the Omicron variant also emphasizes the importance of combining vaccination with individual prevention measures, such as masks, hand washing and social distancing [89].

## 6. The Effect of Smoking and Alcohol Use on Influenza A and COVID-19

Previous studies have implicated the adverse impact of tobacco dependence and heavy drinking on influenza A infections. As reported, a higher risk of hospital admissions after influenza infections might occur in smokers [95,96]. In addition, chronic alcohol use can increase the severity of influenza virus infections by impairing the immune function of the lungs [97,98]. Likewise, increasing data have suggested that unhealthy mental behaviors might be associated with the increased incidence and poor outcomes of COVID-19.

### 6.1. COVID-19 and Tobacco Abuse

Some published studies have discussed that tobacco smoke may affect the outcomes of COVID-19, indicating that smokers were at an increased risk of infection, hospitalization, severe disease and death from COVID-19 [99,100,101,102,103,104,105]. ACE2 and TMPRSS2 receptors were utilized by SARS-CoV-2 to gain entry to host mucosa and replicate in nasal and bronchial epithelial respiratory cells. Both of them were over-expressed in smokers and former smokers [106,107,108], which was recognized as a contributing factor associated with the increased susceptibility to COVID-19 infection [99]. Furthermore, a meta-analysis by Stanton et al. assessed 19 peer-reviewed papers with a total of 11,590 patients with COVID-19. The results suggested that smokers were more likely to experience the progression of COVID-19 than non-smokers, resulting from the adverse effects of smoking on pulmonary immune function [101]. Increasing evidence suggested that cigarette smoke decreased the immune defensive function of respiratory cells [109], with the occurrence of peribronchitis, fibrosis and the damage of airway epithelium [110]. As a result, smokers were more vulnerable to COVID-19 pneumonia, as well as a higher prevalence of complications [102,106,111,112]. In addition, tobacco exposure resulting in poor outcomes of COVID-19 probably was related to worsening the inflammatory process of COVID-19 infection [112]. This view was also supported by the data from a clinical study on 200 COVID-19 patients, which reported the higher level of neutrophil counts, C-reactive protein and neutrophil–lymphocyte ratio in current smokers and former smokers [113]. Notably, smoking habit also was recognized as an additional risk factor for the lower level of antibodies after COVID-19 vaccination [114]. On the other hand, an observational study, including 17,666 COVID-19 patients aged 20–89 years in Japan suggested that the poor progression of COVID-19 was not connected with smoking itself, but the smoke-related comorbidities [115]. Due to the fear of the poor outcomes of COVID-19 infection caused by smoking behavior, an increase in the number of smokers trying to reduce or quit smoking has been observed during the COVID-19 pandemic [116,117].

### 6.2. COVID-19 and Alcohol Addiction

With the outbreak of COVID-19 in December 2019, many countries adopted urgent lockdown measures to reduce the spread of SARS-CoV-2. During lockdown, there was plenty of coverage of the pandemic crisis, including the increased number of new COVID-19 infections and deaths from critical COVID-19, which raised fears of unknown disease around the world. Furthermore, long-term social isolation, unemployment and economic recession from COVID-19 and lockdown measures have an adverse impact on people’s mental health, including increased risk of anxiety, depressive mood, fear, boredom and psychological pressures [118,119,120,121]. All of these mental problems can result in excessive alcohol use [122,123,124,125], which is widely seen as a response to stress [126]. As reported, an increase in alcohol consumption has been observed during the COVID-19 pandemic [123,125,127]. The data from a cross-sectional survey of American adults recruiting 832 participants have shown that a third of participants (34.1%) admitted at least one binge drinking day compared to pre-COVID-19 (26.2%) [128] and sixty percent of participants reported increased alcohol use [123]. Additionally, COVID-19 lockdown may cause a relapse of alcohol use disorders (AUD) or high-risk drinking, which will increase the incidence of alcohol-related adverse consequences, with long-term bad effects on social health [124,129,130].

It is not clear whether alcohol use plays a role in the outcomes of COVID-19 pneumonia. Previous studies suggested that alcohol consumption was not significantly associated with COVID-19 severity and hospitalization [131,132]. However, a recent retrospective cohort study involving 44 centers of the National COVID Cohort Collaborative indicated that a higher prevalence of hospitalization and higher mortality have been found in COVID-19 patients with AUD. They had more comorbidities, including cirrhosis, hypertension and chronic obstructive pulmonary disease (COPD) compared to those without AUD [133]. It is well known that these comorbidities can adversely affect the outcomes of COVID-19. The mechanism underlying the link between alcohol use and COVID-19 infections is still elusive but plausible [134]. Notably, heavy drinking has been proved to affect pulmonary innate immunity and increase airway inflammation, such as excessive cytokine production, thereby reducing defense against COVID-19 infection [135]. In addition, chronic alcohol abuse is an additional risk factor for developing acute respiratory distress syndrome (ARDS), which is associated with critical COVID-19 [136].

## 7. Leftover Effects for the Influenza A and COVID-19

Undoubtedly, seasonal influenza adds up to a huge burden of global disease, with highly cumulative morbidity and mortality worldwide, especially for the elderly and children under five years [137]. The history of influenza pandemics has shown that a new one will occur inevitably at some point in the future. It is still not possible to predict the timing or the severity of next influenza pandemic, but the improvement of global preparedness is so crucial to reduce the deaths and social disruption caused by the next pandemic. Notably, increased efforts for global influenza surveillance are necessary for us to detect the emergence of novel viruses with pandemic potentials. Furthermore, the development of broadly protective influenza vaccines capable of inducing cross-protective and lasting immunity against different influenza viruses is also considered as a priority to strengthen the capacity of public health systems [138].

What is worse is that the COVID-19 pandemic remains far from over, and is leading to lasting effects on different aspects of society, including lifestyle, communication, healthcare, public health, economy, education, etc. First, wearing a mask is the most essential need for a long time. Meanwhile, most schools adopted close management, which decreased the risk of spreading of the virus in schools and super-spread events. However, this measure can have an adverse effect on the children and the youth in terms of physical and mental health. Additionally, in order to control the rapid international spread of the virus, a sharp decline has been shown in the amount of international travel and international flights. In addition, manufacturing shutdown has caused an unavoidable global economy recession, requiring a long recovery period. During the COVID-19 crisis, the large-scale unemployment has caused high stress for middle-class families and vulnerable groups around the world, which can aggravate potential social inequality [139]. On the other hand, for the front-line medical workers, the risk of exposure to the newly confirmed cases will remain for a long time. Furthermore, during the COVID-19 pandemic, medical care was focused on patients infected with SARS-CoV-2; primary and social care were mainly provided by telephone, which might lead to an aggravation of the condition in patients living with mild cognitive decline and dementia [140].

## 8. Co-Management of Seasonal Influenza and COVID-19

For the coming 2023–2024 influenza season, continued transmission of SARS-CoV-2 will raise a great challenge to healthcare resources and concerns about the overall severity of the next influenza. Therefore, enhanced preparedness to reduce the overall burden of influenza and COVID-19 infections is necessary for us to cope with the co-occurrence of seasonal influenza and COVID-19. The specific measures of the combined administration against the two different diseases include availability of rapid diagnostics, rational therapeutic regimens and increased combined vaccination against influenza and COVID-19. Many clinical trials are aiming at co-management of influenza and COVID-19. As of middle December 2022, we downloaded more than 100 clinical trials about the co-administration of influenza and COVID-19 from the three major registries [141]. After removal of duplicates, 57 of them were sorted out and classified by diagnostic, therapy and prevention (Table 2). Thirteen of them (22.8%) were designated to booster the development of the diagnostics of influenza and COVID-19. Twelve (21%) were associated with therapeutic methods and two (3.5%) were involved in drug prophylaxis. In addition, 24 of them (42%) evaluated the safety and immunogenicity of co-administration of the influenza and COVID-19 vaccines.

Only few studies have obtained results and five of which have published the data. A German trial proposed a new rapid test using ion mobility spectrometry coupled with a multicapillary column (MCC-IMS) which could quickly detect influenza A and COVID-19 infections through the breath, providing a fast and non-invasive method for screening of infectious pathogens [142,143]. Another study targeted the extracorporeal membrane oxygenation (ECMO) for ARDS related to influenza and COVID-19. The results indicated that unadjusted 60-day mortality after ECMO initiation was higher in patients infected by COVID-19 than influenza A, probably due to older age and longer hospital stay in COVID-19 patients [144]. In addition, the initial results from a USA clinical trial supported the co-administration of a high-dose quadrivalent influenza vaccine (QIV) and the third dose of SARS-CoV-2 mRNA vaccine in adults. The study findings showed that 22 days after vaccination, unsolicited adverse events were reported for 17.0% of participants in the co-administration group, 14.4% of participants in the SARS-CoV-2 mRNA vaccine group and 10.9% of participants in the QIV group. No serious adverse events or deaths occurred in the three groups. In addition, among the three groups, few differences have been observed in the geometric mean titre (GMT) of the neutralizing antibodies against influenza A and SARS-CoV-2 [146]. Similarly, a Chinese study recruiting 1152 participants also indicated that the seroconversion rate and GMT of SARS-CoV-2 neutralizing antibodies in the co-administration group was similar to those in the SARS-CoV-2 vaccine group. The results suggested that the co-administration of the inactivated SARS-CoV-2 vaccine and QIV is safe and reliable [147]. Such findings indicated that expanding vaccination coverage via reducing vaccine hesitation and friendly reminders is important for us to prepare for the flu season during the COVID-19 pandemic.

## 9. Conclusions

During the past two years, the reports of the COVID-19 have been updated continuously. The current information indicates that SARS-CoV-2 may mutate more frequently than the influenza virus. In addition, we summarized the ongoing clinical trials on co-intervention against COVID-19 and influenza, intending to provide a better understanding of co-containing the two pandemics.

## Figures and Tables

**Table 1:** The comparison of the characteristics of influenza A virus and SARS-CoV-2.

Features	Influenza A Virus	SARS-CoV-2
Timing of the first appearance	1918	December, 2019
Name of pandemic	1918 flu pandemic	COVID-19
Confirmed cases	About 0.5 billion	Over 0.6 billion
Death toll	Over 50 million [6]	Over 6.5 million [8]
Species of virus	The family Orthomyxoviridae	The family of Coronaviridae (genus β-CoVs)
Structure	An enveloped, negative-sense and single-stranded RNA virus [9]	An enveloped, positive-sense and single-stranded RNA virus [10]
Genome size	13.5 kb	29.9 kb
Types of mutations	Genetic reassortment, point mutations [11]	Point mutations
Natural reservoir	Wild waterfowl [12]	Unclear (might be bats) [13]
Mode of transmission	Droplet, aerosol, direct contact and “fecal-oral” route	Droplet, aerosol, direct contact and “fecal-oral” route
Vulnerable population	Young healthy adults (12–17 y) [14]	The elderly and those with comorbidity [15]
Incubation period	1–7 days	2–14 days (a maximum of 24 days) [16]
Host receptor utilized to entry host cells	The terminal glycosides of sialic acid [17]	ACE2 and TMPRSS2 [18]
The protein binding to host receptor	HA	The spike protein
The target for neutralizing antibodies	HA and NA [19]	RBD and NTD of the spike protein [20]
Hematological parameters	Lymphopenia, CRP ↑ [21]	Type I interferon ↓, neutrophil counts ↑ and significant lymphopenia [22]
The main variants	H2N2, H3N2, 2009 H1N1	VOCs (alpha, beta, gamma, delta, omicron)

RNA: ribose nucleic acid; ACE2: angiotensin converting enzyme 2; TMPRSS: transmembrane serine proteases; HA: hemagglutinin; NA: neuraminidase; RBD: receptor-binding domain; NTD: amino-terminal domain; CPR: C-reactive protein; VOCs: variants of concern.

**Table 2:** Clinical trials on combined administration against COVID-19 and influenza.

Type of Intervention	Method	Mechanism	Phase	Age (Years)	Enrollment	Status	Country	Trails Number [ref]
**Diagnostics**								
Diagnostic test	Panbio™ COVID-19/ Flu A&B Rapid Panel	Lateral flow test: antibodies with gold nanoparticles attach to any SARS-CoV-2 antigens present in the sample	n/a	Child, adult, older adult	2472	Completed	United States	NCT05163730
			n/a	14–100 (child, adult, older adult)	665	Recruiting	United States	NCT05354115
			n/a	Child, adult, older adult	1531	Not yet recruiting	n/a	NCT05630365
	Antigen test	Antigen–antibody interaction	n/a	Child, adult, older adult	440,000	Completed	n/a	NCT05087355
	qRT-PCR	Fluorescent reporter sequence-specific DNA probes	n/a	≥18 (adult, older adult)	2000	Enrolling by invitation	Belgium	NCT04527614
	Thoracic CT	Measuring X-ray attenuations by different tissues inside the body	n/a	18–80 (adult, older adult)	200	Recruiting	Mexico	NCT04499378
			n/a	18–80 (adult, older adult)	483	Completed	n/a	NCT04933994
			n/a	18–80 (adult, older adult)	100	Unknown status	Mexico	NCT04497311
	HRCT	Maximize spatial resolution: a narrow slice width (usually 1–2 mm) and a high spatial resolution image reconstruction algorithm are used, field of view is minimized so as to minimize the size of each pixel	n/a	4–80 (child, adult, older adult)	130	Completed	Egypt	NCT04433039
	Chest X-rays and performing AI algorithms on images	Convolutional neural networks	n/a	Child, adult, older adult	200	Unknown status	United Kingdom	NCT04313946
	Serology test	Quantifying IgA and IgG from plasma and/or serum using ELISA	n/a	18–60 (adult)	128	Active, not recruiting	United Kingdom	NCT04568044
	COVID-19 antigen and antibody tests, and influenza rapid test	Antigen–antibody interaction, detecting IgM and IgG markers	n/a	≥18 (adult, older adult)	686	Active, not recruiting	United States	NCT04682132
	MCC IMS	Cluster analysis of nasal air samples	n/a	≥18 (adult, older adult)	76	Completed	Germany	NCT04282135 [142,143]
n/a	Characteristic comparison of ARDS	Retrospective comparison	n/a	≥18 (adult, older adult)	157	Completed	France	NCT04941092
**Therapies**								
Biological	LMSCs	Immunomodulatory effects: regulating innate and specific immune cells and reducing inflammatory response	1	≥18 (adult, older adult)	70	Recruiting	United States	NCT04629105
Drug	AVM0703 (Dexamethasone sodium phosphate)	Suppressing late-stage interferon type I programs in severe COVID-19 patients	1	≥18 (adult, older adult)	16	Not yet recruiting	n/a	NCT04366115
	DAS181 (Sialidase fusion protein)	Protecting human airway epithelium against influenza virus infection	2	≥18 (adult, older adult)	280	Unknown status	China	NCT04298060
	ACE inhibitor	Decreasing the levels of ACE2 in cells might help in fighting COVID-19 infection	n/a	≥18 (adult, older adult)	1,302,508	Unknown status	n/a	NCT04322786
	ACE inhibitor/ARB		n/a	Child, adult, older adult	2574	Recruiting	Spain	NCT04367883
Dietary Supplement	Ayurveda	Traditional herbal supplement: supportive care	n/a	18–60 (adult)	32	Completed	United Kingdom	NCT04351542
			n/a	18–60 (adult)	18	Completed	United Kingdom	NCT04345549
	ABBC1 (symbiotic combination of Beta-glucans and selenium- and zinc-enriched probiotics)	Train immunity: avoiding harmful overreactions after vaccination	n/a	≥18 (adult, older adult)	72	Completed	Spain	NCT04798677
Device	ECMO	Cardiopulmonary bypass: temporarily drawing blood from the body to allow artificial oxygenation of the red blood cells and removal of carbon dioxide	n/a	≥18 (adult, older adult)	100	Completed	n/a	NCT05353725
			n/a	18–80 (adult, older adult)	308	Completed	Italy	NCT05080933 [144]
Behavioral	Healthy lifestyle	Cross-sectional survey	n/a	18–99 (adult, older adult)	1287	Completed	Germany	NCT04653727 [145]
Dietary Supplement	Vitamin D	Reducing the replication of viruses, inducing the expression of ACE2, regulating the immune system	n/a	18–90 (adult, older adult)	41	Terminated	Mexico	NCT04810949
			n/a	≥18 (adult, older adult)	877	Active, not recruiting	United States	NCT04596657
**Prevention**								
Behavioral	Reminder	Reducing vaccine hesitancy	n/a	≥18 (adult, older adult)	97,000	Recruiting	United States	NCT05586165
	Nudge		n/a	≥6 Months (child, adult, older adult)	4152	Recruiting	Australia	NCT05613751
	Persuasion		n/a	18–51 (adult)	3245	Completed	United States	NCT04160975
	VEPMP intervention		n/a	≥65 (older adult)	0	Withdrawn	United States	NCT04761692
n/a	Observing natural history of systemic and nasal mucosal immunity after vaccination	Prospective cohort study	n/a	≥18 (adult, older adult)	150	Recruiting	United States	NCT04794829
	Measuring vaccine effectiveness		n/a	≥18 (adult, older adult)	2400	Not yet recruiting	France	NCT05582239
Vaccination	Flu-MMR vaccines	Stimulating the body’s adaptive immunity	1	≥19 (adult, older adult)	2000	Recruiting	Brazil	NCT05401448
	Bivalent BNT162b2 /qIRV (22/23)/QIV		1	18–64 (adult)	180	Not yet recruiting	n/a	NCT05596734
	influenza and COVID-19 combination vaccine		1, 2	50–70 (adult, older adult)	642	Completed	Australia	NCT04961541
	mRNA-1073 (COVID-19/influenza) vaccine			18–75 (adult, older adult)	550	Active, not recruiting	United States	NCT05375838
	High-dose QIV/COVID-19 mRNA vaccine		2	≥65 (older adult)	278	Completed	United States	NCT04969276 [146]
	qNIV vaccine/SARS-CoV-2 rS vaccine/Matrix-M™ adjuvant		2	50–80 (adult, older adult)	2300	Not yet recruiting	Australia	NCT05519839
	Tetravalent influenza virus lysis vaccine/recombinant new coronavirus vaccine (CHO cell) group		3	≥18 (adult, older adult)	300	Recruiting	China	NCT05107375
	SARS-CoV-2 mRNA vaccine/QIV		3	≥60 (adult, older adult)	0	Withdrawn	n/a	NCT04848467
	Ad26.COV2.S/influenza vaccine		3	≥18 (adult, older adult)	861	Active, not recruiting	United States	NCT05091307
	BNT162b2/SIIV		3	18–64 (adult)	1134	Completed	Australia	NCT05310084
	HZ su/QIV/mRNA-1273		3	≥18 (adult, older adult)	1546	Active, not recruiting	United States	NCT05047770
	Ad26.COV2.S and influenza vaccines		3	≥18 (adult, older adult)	1100	Ongoing	Netherlands	2021–003953-43
	Influenza and COVID-19 combination vaccine		4	≥18 (adult, older adult)	3000	Completed	China	NCT05499351
	COVID-19 inactivated vaccine/QIV/PPV23		4	≥18 (adult, older adult)	3000	Recruiting	China	NCT05298800
	SARS-CoV-2 inactivated vaccine and QIV		4	18–59 (adult)	480	Completed	China	NCT04801888
	COVID-19 vaccine and PPV23/IIV4		4	≥18 (adult, older adult)	1133	Completed	China	NCT04790851 [147]
	Combined immunization of COVAX and PPV23/IIV4		4	≥18 (adult, older adult)	1404	Active, not recruiting	China	NCT05079152
	COVID-19 vaccine/IIV4 + PPV23		4	≥18 (adult, older adult)	1200	Recruiting	China	NCT05480436
	mRNA COVID-19/IIV4		4	≥12 (child, adult, older adult)	450	Recruiting	United States	NCT05028361
	Influenza vaccination		4	≥65 (older adult)	200	Recruiting	Russian Federation	NCT05232292
	COVID-19 vaccine and influenza vaccine		5	≥18 (adult, older adult)	160	Completed	Netherlands	2021–002186-17
	influenza, SARS-CoV-2 and pneumococcal vaccinations		6	≥18 (adult, older adult)	1000	Ongoing	Austria	2021–002789-42
	Influenza and COVID-19 combination vaccine		n/a	18–60 (adult)	1500	Recruiting	Australia	NCT05110911
	Flu vaccine		n/a	18–64 (adult)	37	Completed	United States	NCT04579588

ref: completed study published; n/a: not applicable; qRT-PCR: reverse transcription-polymerase chain reaction; DNA: deoxyribonucleic acid; CT: computed tomography; Ig: immunoglobulin; ELISA: enzyme-linked immunosorbent assay; AI: artificial intelligence; HRCT: high-resolution computed tomography; MCC IMS: multicapillary ion mobility spectrometry; ARDS: acute respiratory distress syndrome; LMSCs: longeveron mesenchymal stem cells; ACE: angiotensin converting enzyme; ARB: angiotensin receptor blockers; ECMO: extracorporeal membrane oxygenation; MMR: measles, mumps and rubella; qIRV: modRNA quadrivalent influenza vaccine; qNIV: quadrivalent hemagglutinin nanoparticle influenza vaccine; rS: recombinant spike; QIV: quadrivalent influenza vaccine; HZ su: herpes zoster subunit; PPV23: 23-valent pneumococcal polysaccharide vaccine; IIV4: quadrivalent inactivated influenza vaccine; COVAX: inactivated SARS-CoV-2 vaccines (vero cell); SIIV: seasonal inactivated influenza vaccine.

## Data Availability

No new data were created or analyzed in this study. Data sharing is not applicable to this article.
